# Comparative genomic analysis of *Tropheryma whipplei *strains reveals that diversity among clinical isolates is mainly related to the WiSP proteins

**DOI:** 10.1186/1471-2164-8-349

**Published:** 2007-10-02

**Authors:** My-Van La, Nicolas Crapoulet, Pascal Barbry, Didier Raoult, Patricia Renesto

**Affiliations:** 1 Unité des Rickettsies, CNRS-UMR6020, IFR48, Faculté de Médecine, 27 Bd Jean Moulin, Marseille, F13385, France; 2 CNRS, Institut de Pharmacologie Moléculaire et Cellulaire, UMR6097, Sophia Antipolis, F06560, France; 3 Université de Nice Sophia Antipolis, Institut de Pharmacologie Moléculaire et Cellulaire, UMR6097, Sophia Antipolis, F06560, France

## Abstract

**Background:**

The aim of this study was to analyze the genomic diversity of several *Tropheryma whipplei *strains by microarray-based comparative genomic hybridization. Fifteen clinical isolates originating from biopsy samples recovered from different countries were compared with the *T. whipplei *Twist strain. For each isolate, the genes were defined as either present or absent/divergent using the GACK analysis software. Genomic changes were then further characterized by PCR and sequencing.

**Results:**

The results revealed a limited genetic variation among the *T. whipplei *isolates, with at most 2.24% of the probes exhibiting differential hybridization against the Twist strain. The main variation was found in genes encoding the WiSP membrane protein family. This work also demonstrated a 19.2 kb-pair deletion within the *T. whipplei *DIG15 strain. This deletion occurs in the same region as the previously described large genomic rearrangement between Twist and TW08/27. Thus, this can be considered as a major hot-spot for intra-specific *T. whipplei *differentiation. Analysis of this deleted region confirmed the role of WND domains in generating *T. whipplei *diversity.

**Conclusion:**

This work provides the first comprehensive genomic comparison of several *T. whipplei *isolates. It reveals that clinical isolates originating from various geographic and biological sources exhibit a high conservation rate, indicating that *T. whipplei *rarely interacts with exogenous DNA. Remarkably, frequent inter-strain variations were dicovered that affected members of the WiSP family.

## Background

*Tropheryma whipplei *is a Gram positive bacterium responsible for Whipple's disease [1]. This chronic, multisystemic infection is mainly characterized by intestinal malabsorption, but also involves other organs such as the heart and central nervous system and is ultimately fatal without appropriate treatment [2]. *T. whipplei *infection in human beings is particularly interesting due to the wide range of disease outcomes [3]. While it is known to be associated with the environment [4,5], the natural reservoir of *T. whipplei *is still unknown.

In 2000, the first human isolate of this bacterium was successfully cultured in a fibroblast cell line [6]. This allowed investigators to examine the phenotypic characteristics of the bacterium, about which little had been known [1,7], and made possible the sequencing of the 0.93 Mb genome [8,9]. This major achievement has provided new perspectives on both the diagnosis and treatment of Whipple's disease [10,11]. Among the first post-genomic applications was the successful design of a comprehensive culture medium, based on computer modeling of metabolic networks, which allowed the axenic growth of the microorganism [12]. The use of the axenic culture medium for diagnostic purposes has enabled isolation of several *T. whipplei *strains from cerebrospinal fluid, blood, synovial fluid, lymph node, cardiac valve, skeletal muscle, and stool [3]. *T. whipplei *replication in the absence of eukaryotic cells has also facilitated the immunoproteomic analysis of this pathogen, which was conducted using sera from patients [13]. Finally, the absence of eukaryotic nucleic acids favored optimization of RNA extraction [14], thus making microarray-based transcriptional studies possible [15,16].

The DNA microarrays allow for the identification of changes in gene expression in response to specific stimuli from the environment on a global scale. This technology also provides a powerful tool to study genome variability among strains. Thus, microarray-based comparative genomic hybridization (CGH) was described as a good alternative to whole genome sequencing [17], and has been applied for several human pathogens [18]. Such an approach was shown to be useful to assess genome plasticity and evolutionary trends including analysis of potential gene transfer events [19]. Moreover, CGH analysis was found to be helpful in revealing gene polymorphisms associated with distinct tissue tropisms or niche adaptation [20-22], in characterizing virulence factors [23-28] and in providing putative vaccine candidate antigens [23,29].

To date, two *T. whipplei *strains have been sequenced, TW08/27 [9] and Twist [8]. These strains were isolated from cerebrospinal fluid collected in Germany [9] and the cardiac valve of a Canadian patient [6], respectively. A comparative analysis of the two strains revealed a large chromosomal inversion. This observation, associated with a high nucleotide identity between both strains (> 99%), was considered to be an indication of a very active genome rearrangement process [8]. Such genomic recombination was hypothesized to be mediated by the WND protein-coding repeats within genes encoding the WiSP membrane proteins and to be associated with changes in the set of proteins exposed at the surface of the bacteria. According to this perspective, recombination would constitute an adaptive response to the host defense or to environmental conditions.

Due to the variable pathological profiles which can result from *T. whipplei *infection, we hypothesized that it could be of interest to further define the genetic diversity of various strains. To obtain information on *T. whipplei *gene content, we performed a comparative analysis of sixteen clinical isolates by CGH using the whole *T. whipplei *Twist strain cDNA microarray. Results were subsequently validated by PCR and sequencing.

## Results

### Accuracy of genomic DNA hybridization on *T. whipplei *microarray

Screening for divergent or absent regions in *T. whipplei *strains isolated in our laboratory was carried out using a microarray chip containing PCR products from 804 genes corresponding to 99.5% of the *T. whipplei *Twist genome [15]. In the first step, the accuracy of the *T. whipplei *genomic DNA hybridization assays was determined through control experiments self-hybridizing Cy5-labeled Twist with Cy3-labeled Twist. As expected, the corresponding GACK analysis, which allows coding sequence (CDS) classification as either conserved or absent/divergent using a trinary output under stringent conditions [30], showed that 100% of the CDSs were present. No hybridization was observed against negative control DNAs present on the microarrays. Accordingly, we concluded that both experimental and computational processing were appropriate for analyzing our data.

### Global analysis of conserved and divergent CDSs in the *T. whipplei *genome

**Figure 1:**
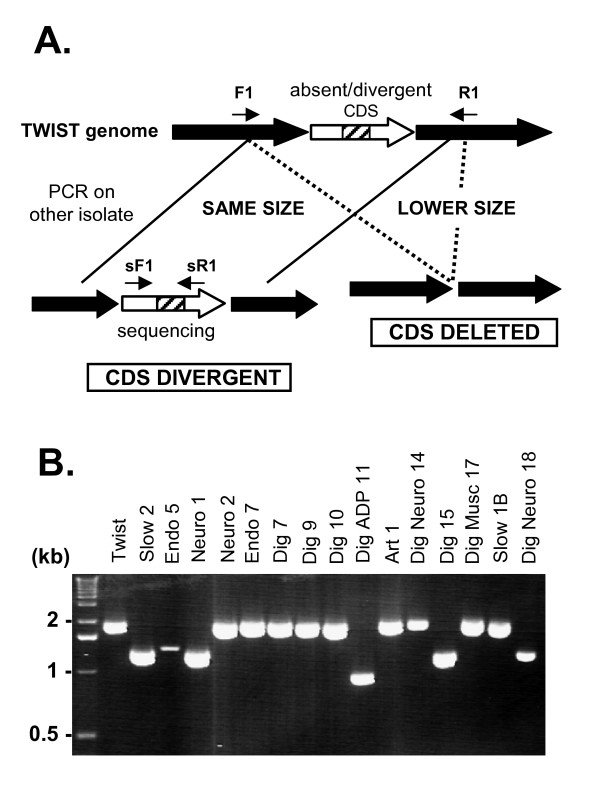
**(A) Schematic representation of PCR and sequence-based strategies used to further investigate genomic deletion or divergence**. Two couples of primers were designed based on the genome sequence of *T. whipplei *Twist strain, including one (F1/R1) flanking the gene predicted absent/divergent by CGH analysis, and another (sF1/sR1) flanking the PCR amplicon spotted on the microarray represented by the hatched square. **(B) PCR analysis of a putative TWT596 deletion on various *T. whipplei *strains**. The amplicon size obtained using the primers TWT595F1 and TWT597R1 and *T. whipplei *Twist DNA as positive control was of 2040 nt. Lower size amplicons were obtained with Slow2, Endo5, Neuro1, DigADP11, Dig15 and DigNeuro18, indicating that the gene was deleted in these strains. The first lane corresponds to DNA size standard (1 kb DNA ladder, Invitrogen).

**Figure 2:**
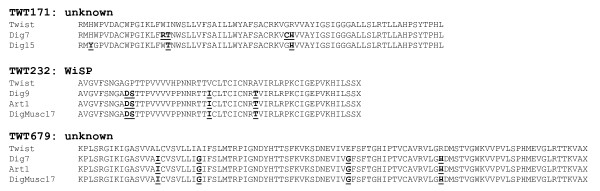
**Alignment of partial protein sequences from several *T. whipplei *isolates**. Divergent amino acids relative to *T. whipplei *Twist strain were underlined and written in bold characters.

**Figure 3:**
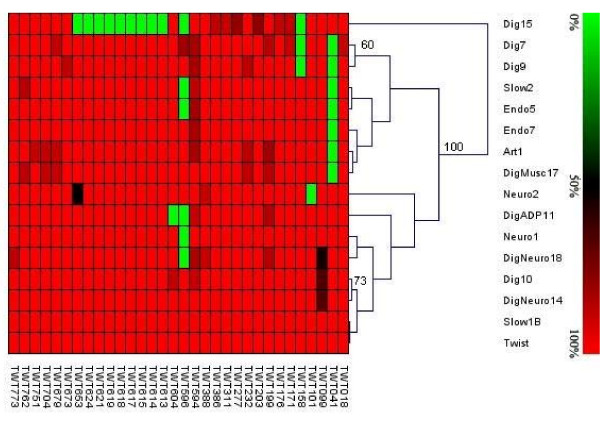
**Phylogenic relationships of *T. whipplei *isolates based on CGH data**. CGH data were analyzed using TIGR Multiexperiment Viewer (MeV) [51]. Statistical analysis of each node of the tree was determined by bootstrap analysis with 1,000 replicates using the clustering support tree. Only bootstrap values greater than 50 are indicated. In the color scheme at the top of the figure, the brightest green corresponds to CDSs that are absent and brightest red indicates CDSs that are absent. The graduate scale corresponds to the percent of homologies of sequenced genes with Twist genes.

Given the estimated probability of CDS presence deduced from the GACK analysis of the 45 hybridization profiles obtained, it appeared that *T. whipplei *genomic sequences were highly conserved across the 16 isolates tested (Table 1). These 45 hybridizations consisted of three repeats of the hybridization of 15 distinct *T. whipplei *isolates *vs*. the Twist strain. Comparative analysis showed that relative to the Twist isolate, the percentage of conserved CDSs ranges from 97.76% (strain Dig15) to 99.88% (strains Slow1B, Neuro1, and DigNeuro14). The heat map visualization of genomic variations showed that there were differences spread across the entire genomes (Additional file 1). Only 34 genes from the Twist isolate were predicted to be absent or highly divergent in at least one tested strain. Reduced hybridization can result either from whole gene deletion or from nucleotide sequence variation. In order to clarify the nature of the observed deletion/divergence, PCR were performed using specific primers located upstream and downstream of the targeted CDSs. The electrophoresis migration profile obtained for TWT596 is shown in Figure 1. Analysis of the 72 deletions/divergences showed a reduced PCR amplicon size consistent with the gene deletion observed in the 27 cases (Table 2). Sequencing of the PCR products with the same migration profile as those obtained from the *T. whipplei *Twist strain indicated that the lower hybridization resulted from a sequence variation in 43/45 genes. Further analysis of nucleotide changes resulted in the identification of one silent mutation (TWT176 in Dig15 strain). The genes were interrupted by STOP codons in 9 cases, and 3 of these were related to TWT099, which encodes a protein of unknown function. The observed sequence divergences mainly induce amino acid changes. Interestingly, when a gene exhibited variations in several strains, the amino acid changes they trigger were found to be partly conserved (Figure 2 and Additional file 2). In two cases of genes encoding proteins of unknown function, TWT151 and TWT722, the predicted deletion/divergence was not associated with nucleotide changes. These false-positive values were not included in the dendrogram established from the overall CGH assay including sequencing data. As shown in Figure 3, Dig7, Dig9, Slow2, Endo5, Endo7, Art1 and DigMus17 formed a group distinct from Neuro2, DigADP11, Neuro1, DigNeuro18, Dig10, DigNeuro14, Slow1B; and Twist. Dig15 appeared to be phylogenically distinct from all other isolates.

**Table 1:** *T. whipplei *isolates used in this study and percentage of conserved CDSs identified through CGH analysis

**Isolate designation**	**Sample origin**	**Clinical manifestations**	**Patient (sex, age)**	**Geographical origin**	**% conserved CDSs**
Twist	Aortic valve	EW	M42	Canada	100.00
Endo 5	Heparinized blood	EW	M61	France	99.63
Endo 7	Aortic valve	EW	M67	Portugal	99.75
Slow 1B	Faeces	CWD, digestive relapse	F34	France	99.88
Slow 2	Duodenal biopsy	CWD	F70	France	99.50
ART 1	Synovial fluid	CWD	M68	France	99.13
Neuro 1	Cerebrospinal fluid	CWD, neurologic relapse	M57	Germany	99.88
Neuro 2	Cerebrospinal fluid	CWD with neurologic involvement	F40	France	99.63
Dig 7	Heparinized blood	CWD	M64	France	99.00
Dig 9	Heparinized blood	CWD	M35	France	99.38
Dig 10	Cerebrospinal fluid	CWD with neurologic involvement	M46	Germany	99.63
Dig ADP 11	Mesenteric lymph node	CWD	M56	France	99.50
Dig Neuro 14	Cerebrospinal fluid	CWD with neurologic involvement	M50	Germany	99.88
Dig 15	Cerebrospinal fluid	CWD with neurologic involvement	M60	Germany	97.76
Dig Musc 17	Muscle	CWD	F73	France	99.25
Dig Neuro 18	Cerebrospinal fluid	CWD with neurologic involvement	M68	France	99.25

CWD = Classic Whipple's disease

EW = Endocarditis due to *T. whipplei*

M: male; F: female

**Table 2:** PCR and sequencing confirmation of CDSs identified as absent/divergent by CGH analysis

				**PCR and sequencing**		
				
**Gene ID**	**Gene name**	**Gene product**	**Intensity of Hybridization lower than with Twist**	**Genes deleted**	**Genes with nt variation(s)**	**Genes conserved**
TWT018	*deoD*	purine nucleoside phosphorylase	1		1	
TWT041	*-*	hypothetical protein	7	7		
TWT099	*-*	hypothetical protein	3		3	
TWT101	*-*	hypothetical protein	1	1		
TWT151	*-*	hypothetical protein	1			1
TWT158	*-*	hypothetical protein	3	3		
TWT171	*-*	hypothetical protein	2		2	
TWT176	*-*	hypothetical protein	1		1	
TWT199	*ftsE*	cell division ATP-binding protein	5		5	
TWT203	*ksgA*	dimethyladenosine transferase	1		1	
TWT232	***-***	**WiSP family protein**	3		3	
TWT277	*-*	hypothetical protein	1		1	
TWT311	*-*	hypothetical protein	1		1	
TWT386	*trpE*	anthranilate synthase component I	1		1	
TWT388	*-*	hypothetical protein	2		2	
TWT594	***-***	**WiSP family protein**	9		9	
TWT596	***-***	**WiSP family protein**	7	6	1	
TWT604	*-*	hypothetical protein	2	1	1	
TWT613	*-*	hypothetical protein	1	1		
TWT614	*ftsK*	cell division protein FtsK	1	1		
TWT615	*-*	hypothetical protein	1	1		
TWT617	*-*	hypothetical protein	1	1		
TWT618	*wblE*	Putative transcription reg. protein	1	1		
TWT619	-	hypothetical protein	1	1		
TWT621	-	hypothetical protein	1	1		
TWT624	**-**	**WND-containing WiSP family protein**	1	1		
TWT653	-	membrane protein	2	1	1	
TWT673	-	hypothetical protein	1		1	
TWT679	-	hypothetical protein	3		3	
TWT704	-	hypothetical protein	2		2	
TWT722	-	hypothetical protein	1			1
TWT751	-	hypothetical protein	1		1	
TWT762	-	hypothetical protein	2		2	
TWT773	-	hypothetical protein	1		1	

		**TOTAL**	**72 **(100%)	**27 **(37.50%)	**43 **(59.70%)	**2 **(2.80%)

The values correspond to the number of strains for which mentioned parameters were found different to that observed in *T. whipplei *Twist. (nt): nucleotide.

### Major genomic changes are related to genes encoding for WiSP family proteins

The distribution of the 34 absent/divergent CDSs was analyzed according to their functional classification. Of these, 23 genes encoded hypothetical proteins. This corresponds to 10% of unknown genes annotated in the *T. whipplei *Twist genome. The functional category exhibiting the most number of genes altered, including both deletions and mutations, corresponds to membrane proteins. Thus, 4/15 proteins (26.7%) in the WiSP family were found to be absent or divergent. While most of the genes (58.9%) exhibited divergence in only 1 of the 16 examined strains, more frequent changes were observed in the WiSP family proteins (Table 2). TWT232 and TWT594 sequence variations were observed in 3 and 9 *T. whipplei *strains, respectively, while TWT596 was found to be absent in 6 strains and divergent in another one. With the exception of Slow1B, DigNeuro14 and Neuro2 strains, the WiSP proteins differed from Twist in all *T. whipplei *strains. Other CDS variations shared by several *T. whipplei *strains encode either hypothetical proteins (9 cases), membrane proteins (1 case), or FtsE, a cell division ATP-binding protein (which had nucleotide changes in the Dig7, DigADP11, Art1, DigMusc17, and DigNeuro18 strains).

### CGH analysis reveals a major deletion in *T. whipplei *DIG15

**Figure 4:**
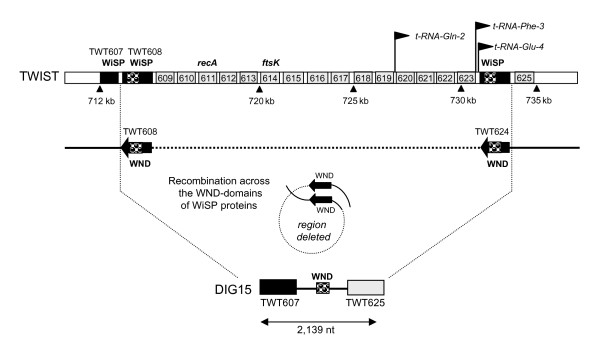
**Schematic representation of the genomic rearrangement observed in DIG15**. Two WND repeats (Accession number: PF07861) were found in the genes TWT608 and TWT624 that are respectively located at both extremities of the 19.3 kb region of the Twist strain deleted in DIG15.

A more detailed analysis was then performed on the genomic region of *T. whipplei *DIG15 strain ranging from TWT608 to TWT624. Several genes consecutively located in this region were predicted to be absent/divergent in this strain, and other genes were characterized as uncertain (Table 3). PCR assays were thus designed to bridge each of the putatively deleted regions. A 2,950 bp amplicon was amplified using forward and reverse primers located on TWT607 and TWT625, and further sequenced to determine the exact location of the deletion event (Additional file 3). These data demonstrated that all 17 CDSs located between TWT607 and TWT625 were absent in *T. whipplei *DIG15. Half of these corresponded to proteins with unknown functions (Table 3). Genes encoding previously characterized proteins such as *recA *and *ftsK *were also absent. The GC% value of this deleted region was 46.59%, compared to 46.3% for the whole genome [8]. Interestingly, the boundaries of this deleted genomic region are two genes that belong to the WiSP membrane protein family, namely TWT608 and TWT624. The sizes of these genes in the *T. whipplei *Twist strain are 1,044 and 2,070 nucleotides, respectively. Both genes share an N-terminal WND domain that could trigger a recombination event. From BLAST analysis, both genes were found to be 99% identical over a 784 bp nucleotide fragment. In the DIG15 sequenced amplicon, the region without BLAST similarities to TWT607, TWT625 or their adjacent intergenic spacer is only 563 nucleotides. This indicates that the recombination event induced a deletion around 19 kb within the WND domain-containing WiSP proteins. This deletion was also observed by PCR in the primary isolate stored after the first passage in the fibrosblast cell line (not shown). This putative recombination event is shown in Figure 4.

**Table 3:** List of genes deleted in DIG15

**Gene ID**	**Gene name**	**CGH**	**Sequencing**
*TWT607*	*WiSP family protein*	*+1*	*present*
TWT608	**WND-domain containing, WiSP family protein**	0	deleted
TWT609	*miaA*; tRNA isopentenylpyrophosphate transferase	0	deleted
TWT610	hypothetical protein	0	deleted
TWT611	*recA*; recombinase A	0	deleted
TWT612	*pgsA*; CDP-diacylglycerol – glycerol-3-phosphate 3-phosphatidyltransferase	0	deleted
TWT613	hypothetical protein	-1	deleted
TWT614	*ftsK*; cell division protein FtsK	-1	deleted
TWT615	hypothetical protein	-1	deleted
TWT616	*thyX*; thymidylate synthase	0	deleted
TWT617	hypothetical protein	-1	deleted
TWT618	*wblE*; putative transcription regulatory protein	-1	deleted
TWT619	ORFan	-1	deleted
TWT620	hypothetical protein	0	deleted
TWT621	hypothetical protein	-1	deleted
TWT622	*rph*; ribonuclease PH	0	deleted
TWT623	hypothetical protein	0	deleted
TWT624	**WND-domain containing, WiSP family protein**	-1	deleted
*TWT625*	*putative serine protease*	*+1*	*present*

(+1): present; (0): uncertain; (-1): absent

## Discussion

One of the major results from the CGH analysis performed with a microarray composed of PCR products amplified from the 0.93 Mb *T. whipplei *Twist genome [15], is the remarkable preservation of the genomic content of the 16 *T. whipplei *isolates. As previously claimed [31,32], one of the limitations of this technique is the failure to detect genes that were not spotted on the microarrays. However, the high genomic conservation rate observed in this study is consistent with the fact that *T. whipplei *gene family size is an ancestral feature. This suggests an evolutionary scenario where the isolates originating from diverse sources have all evolved from a common ancestor.

The percentage of *T. whipplei *Twist probes exhibiting differential hybridization in other strains is in the same range as those described for the *Francisella tularensis *[33], *Chlamydia trachomatis *[22], *Coxiella burnetii *[28], and *Rickettsia prowazekii *species [24]. A common trait among these pathogens and *T. whipplei *is a small genome size between 0.93–2.0 Mb, a marked AT nucleotide bias, and the loss of several metabolic pathways, which explains their complex nutritional requirements [8,34-37]. While *F. tularensis *can replicate under axenic conditions *in vitro*, it is thought that obligate host-dependent survival is required in its natural life cycle [37]. The strictly intracellular niche of *T. whipplei *is still under debate. However, the high level of similarity between *T. whipplei *strains strongly suggests that these microorganisms are mainly associated with host cells *in vivo*. While free-living and facultative intracellular bacteria might be constantly bombarded with foreign genes, genetic exchange opportunities are limited for obligate intracellular bacteria, which may result in few evolutionary events of gene transfer [38,39]. In contrast to what was observed for *T. whipplei *and other host-associated pathogens, the CGH analysis performed on enteric bacteria including *Salmonella enterica *[40], *Campylobacter jejuni *[41], *Helicobacter pylori *[42], *Shigella *[27,31] or *Enterococcus faecalis *[43] evidenced 15 to 23% genetic variations.

Among the 34 lacking or divergent CDSs found in at least one of the tested strains, 23 encode for proteins of unknown function, which can be considered non-essential for bacterial survival and replication. This is mainly the case for TWT041 and TWT099-encoded proteins, which are deleted and interrupted, respectively, in several *T. whipplei *strains. While strain-specific divergences were shown for *deoD *(Dig7), *ksgA *(Dig15), and *trpE *(Dig15), nucleotide changes in *ftsE *were observed in 31.25% of isolates. These modifications lead to amino acid changes in 4 isolates, the gene being interrupted in the fifth one. Alignment of the whole *ftsE *Twist gene [8] with the TW08/27 gene [9] showed 70% similarity, which is in disagreement with the 99% identity of both genomes at the nucleotide sequence level [8]. Recent evidence showed that FtsE was absolutely required for the process of bacterial division in low osmotic strength conditions, because this protein ensures the stability of the septal ring assembly [44]. In contrast, this ABC transporter-type protein was described as non-essential for bacteria grown under high-osmolarity conditions, as shown by viable *ftsE *null mutants [44,45]. Relationships between mutations within the *Aeromonas hydrophila ftsE *gene and the filamentous phenotype of the bacteria, which likely interferes with opsonophagocytosis, were also observed [46]. These authors hypothesized that the formation of large bacterial aggregates that could not be ingested by phagocytic cells could be used to evade the immune system. The relevance of FtsE changes for each *T. whipplei *strains is questionable, but it can be hypothesized that the protein plays a role in adaptating to environmental change.

This CGH analysis showed that the WiSP proteins, heterogeneous surface proteins grouped into a family based on several identifying features including WND repeats [9], are highly divergent across the *T. whipplei *strains compared in this study. A high divergence rate of genes related to surface structure proteins has been described for several pathogens including *Yersinia pestis *[21], *Campylobacter jejuni *[41], and *Shigella *[31]. The heterogeneity of the *T. whipplei *WiSP proteins is consistent with observations made in previous studies using a shotgun sequence assembly of the *T. whipplei *strain TW08/27 [9]. Indeed, these authors provided evidence for the existence of 44 loci located within the WiSP coding sequence TW570, where variations that mainly resulted in single amino acid changes were observed. PCR amplification and sequencing of the N-terminal region of this gene further revealed variations when the same culture was collected at different passages [9]. Comparing different isolates showed that TWT594 and TWT596, which are partly homologous to the TW570 gene of *T. whipplei *TW08/27 strain, either exhibited several amino acid mutations or were lacking. Amino acid changes were observed in the WiSP protein encoded by TWT594 in 8 isolates, and a frame-shift mutation was found in another strain. Deletion of the gene encoding the TWT596 WiSP protein was observed in 43% of the strains analyzed. While 4.2% of the *T. whipplei *CDSs appeared to have diverged in the Twist strain relative to the other 15 strains tested, this value increased to 26.7 % when genes that belong to the WiSP protein family were considered. These data emphasize the diversity of such membrane proteins across various strains. Altogether, these findings suggest that divergent evolution of this select set of genes could be associated with development of bacteria which have similar genomic contents, in different environments. WiSP proteins exhibit amino acid hypervariation that could be responsible for various pathological features and immune system evasion.

There was a notable deletion of 17 CDSs corresponding to 19,052 kb in the *T. whipplei *DIG15 strain, ranging from TWT608 to TWT624. PCR amplification carried out on the primary isolate of DIG15 concluded that this evolutionary event did not occur *in vitro*. As previously reported for the chromosomal inversion seen in the *T. whipplei *Twist and TW08/27 strains [8], this deletion event is associated with flanking WiSP membrane protein family coding genes. This fits well with the proposed role of the conserved N-terminal WND domain in these proteins, with respect to promoting genome recombination [8]. The observed deletion probably results from homologous recombination events across such conserved domains. A correlation between the hypermutable regions of the *F. tularensis *genome and the presence of repeated sequences has been previously reported [33]. The majority of deleted genes in the *T. whipplei *DIG15 strain encode proteins with unknown function that can be considered as non-essential. DNA rearrangements are believed to contribute to the fitness of a pathogen in specific environments. Thus, the adaptation of bacterial pathogens from the *Bartonella *and *Rickettsia *genera, to host-restricted vectors such as lice, was associated with accelerated rates of genome degradation [47]. Interestingly, both major genomic rearrangements, such as the chromosomal inversion among Twist and TW08/27 and the 19 kb deletion in DIG15, are located in the same region which can be considered as the major hot-spot for intra-strain differentiation in *T. whipplei*.

## Conclusion

In summary, this work provides the first comprehensive genomic comparison of several isolates of *T. whipplei*. It reveals that clinical isolates originating from various geographic and biological sources exhibit a high conservation rate, suggesting that *T. whipplei *rarely interacts with exogenous DNA. The frequent variations among members of the WiSP membrane protein family is a remarkable trait. The significance of these changes remains to be investigated.

## Methods

### *T. whipplei *isolates and preparation of genomic DNA

*T. whipplei *isolates (n = 16) used in this study are detailed in Table 1. These strains correspond to the whole collection of *T. whipplei *isolates available in our laboratory when the project was initiated. Of the available strains, 3/16 were isolated from patients with endocarditis, which is representative of Whipple's disease patients worldwide [3]. None of the patients were related to each other or had been in contact. All bacterial isolates used in this study were grown under axenic conditions as previously described [12]. Eighteen day-old bacterial cultures (300 ml) were collected by centrifugation 16,900 × *g *for 10 min at 4°C. The resulting pellets were resuspended in 3 ml PBS and stored at -20 C. DNA was extracted using the QIAamp^® ^DNA mini kit according to the manufacturer's instructions (Qiagen, Courtaboeuf, France), and the DNA concentration was determined by UV absorbance at 260 nm.

### Genomic DNA labeling

In the microarray experiments described below, DNA from the *T. whipplei *Twist strain was used as a reference, whereas the DNA from other isolates was referred as test DNA. DNA was labeled with Cy3-dCTP and Cy5-dCTP (Amersham Biosciences, Piscataway, NJ, USA) for the test and reference samples, respectively, using the BioPrime^® ^Array CGH Genomic Labeling System (Invitrogen, Carlsbad, CA, USA). For each hybridization, 1 μg of *T. whipplei *Twist DNA and test DNA were primed with random octamers, and Cy3- or Cy5-labeled probes were generated under the extension and fluorescent nucleotide incorporation activity of exo-Klenow polymerase. The levels of incorporation were quantified by absorbance measurement at 550 nm and 650 nm. Samples were processed for hybridization on microarrays when the incorporation levels were ≥ 50 pmol of fluorochromes per μg of DNA.

### Microarray hybridization

This study used *T. whipplei *microarrays spotted with 804 DNA fragments amplified from the Twist strain by PCR [15]. These 804 amplicons corresponded to 99.5% of annotated CDSs from the *T. whipplei *Twist genome. These microarrays also included DNA sequences from yeast intergenic regions (Lucidea™ Universal Score Card™, Amersham) that were used as negative controls. Following a post-processing step, 7 μg of labeled DNA for each reference and test sample were pooled for hybridization onto microarrays as previously described [15]. All experiments were conducted in triplicate, yielding 12 independent measurements for each condition (representing 4 technical and 3 biological replicates). After an 18-hour incubation at 42°C, the slides were washed, dried with compressed nitrogen, and scanned with the ScanArray^® ^Express (Perkin Elmer, Boston, MA, USA).

### Microarray data analysis

The signal intensity, local background for both fluorescence channels of each spot, and the preliminary exclusion of irrelevant values, as flagged, were determined from TIF images using the QuantArray^® ^Microarray Analysis Software version 3.0.0.0 (Packard BioScience). Data filtering and normalization were then performed using the Microsoft Excel software. Spots with background-corrected signal intensity (median) less than two-fold of the background intensity (median) in both channels were excluded from further analysis. The background-subtracted signals derived from the remaining spots were normalized by the global median method, and the normalized log ratio of test/reference signal for each spot was recorded. For each CDS and each comparative strain analysis, the median value of 12 normalized log ratios of the test/reference signal was used for GACK analysis [30]. Trinary output was then applied to determine if the CDS was present, uncertain, absent, or divergent. When the deduced estimated probability of presence (EPP) was 100%, genes were designated as present (assigned '+1'). Genes with 0% EPP were assigned into absent/divergent category ('-1'). The lack of hybridization to a probe spot can indeed result from the absence of the gene or it can be due to divergence of the gene within a portion of the gene including the probe. When the EPP values ranged between 0% and 100%, the presence or absence/divergence of the gene remained uncertain. GACK calculates dynamic cut-off values and can generate improved identification of absent/divergent genes independently of any normalization process that would be strongly influenced by differences between the reference strain and tested strains. In our case, because the number of absent/divergent genes is very small, the global median method of normalization is reasonable.

### PCR verification and sequence analysis

To validate the results predicted by CGH, all deletion/divergence events were verified by PCR amplification and/or subsequent sequencing. For every gene suspected to be absent/divergent, one pair of primers was designed to anneal to the upstream and downstream genes flanking the target region. The reaction was performed with DNA from each strain using the Expand High Fidelity PCR (Roche, Penzberg, Germany) according to the manufacturer's protocol. All PCR products were examined using 1% agarose gels and stained with ethidium bromide. When the size of the PCR amplicons was similar to that obtained with *T. whipplei *Twist, the putative divergence in nucleotide sequences was assessed through sequencing (ABI PRISM^® ^BigDye^® ^Terminator v1.1 Cycle Sequencing Kit, Applied Biosystems, Foster City, CA, USA). Obtained sequences were compared to that of the *T. whipplei *Twist strain by the CLUSTALW program [48]. All primers used in this study are listed in Additional file 4.

### Phylogenetic analysis

A numerical score was assigned to each CDS analyzed based on the following criteria: +3 for present, -3 for absent, and +1 for containing nucleotide variation. A dendrogram was constructed using hierarchical clustering with Euclidean distance by Genesis software version 1.7.2 [49].

### Accession numbers

All CGH results are available in the GEO database [50] under accession number GSE7453. Nucleotide sequences correspond to GenBank accession numbers from [GenBank:EF536098] to [GenBank:EF536142].

## Authors' contributions

NC and MVL carried out *T. whipplei *culture, DNA extraction, and microarray hybridization. MVL also performed comparative genomic analysis and sequencing and participated in drafting the manuscript. PB contributed to DNA microarray construction. DR proposed the research goal and provided critical review of the manuscript. PR supervised the design and the coordination of the entire study and wrote the main draft of the manuscript. All authors read and approved the final manuscript.

## Supplementary Material

Additional file 1 Genome composition of 16 *T. whipplei *isolates based on CGH microarray data. This heat map was constructed from the GACK analysis results. Each row corresponds to a specific gene in Twist gene order, whereas columns represent strains analyzed. The CDSs status is color-coded: red, unchanged; green, absent/divergent; black uncertain. The grey lines correspond to missing values. Click here for file

Additional file 2 Alignment of protein sequences from *T. whipplei *isolates analyzed in this study. Click here for file

Additional file 3 Nucleotide sequences of the genomic region amplified in *T. whipplei *DIG15 with primers TWT607F1 and TWT625R1. Click here for file

Additional file 4 Oligonucleotide primers used for PCR and sequencing. Click here for file
