# Supplementary figures and images for: Comparative genomic analysis of Tropheryma whipplei strains reveals that diversity among clinical isolates is mainly related to the WiSP proteins
Source: BMC Genomics. 2007 Oct 2;8:349. doi: 10.1186/1471-2164-8-349 (PMC2078596; doi:10.1186/1471-2164-8-349)

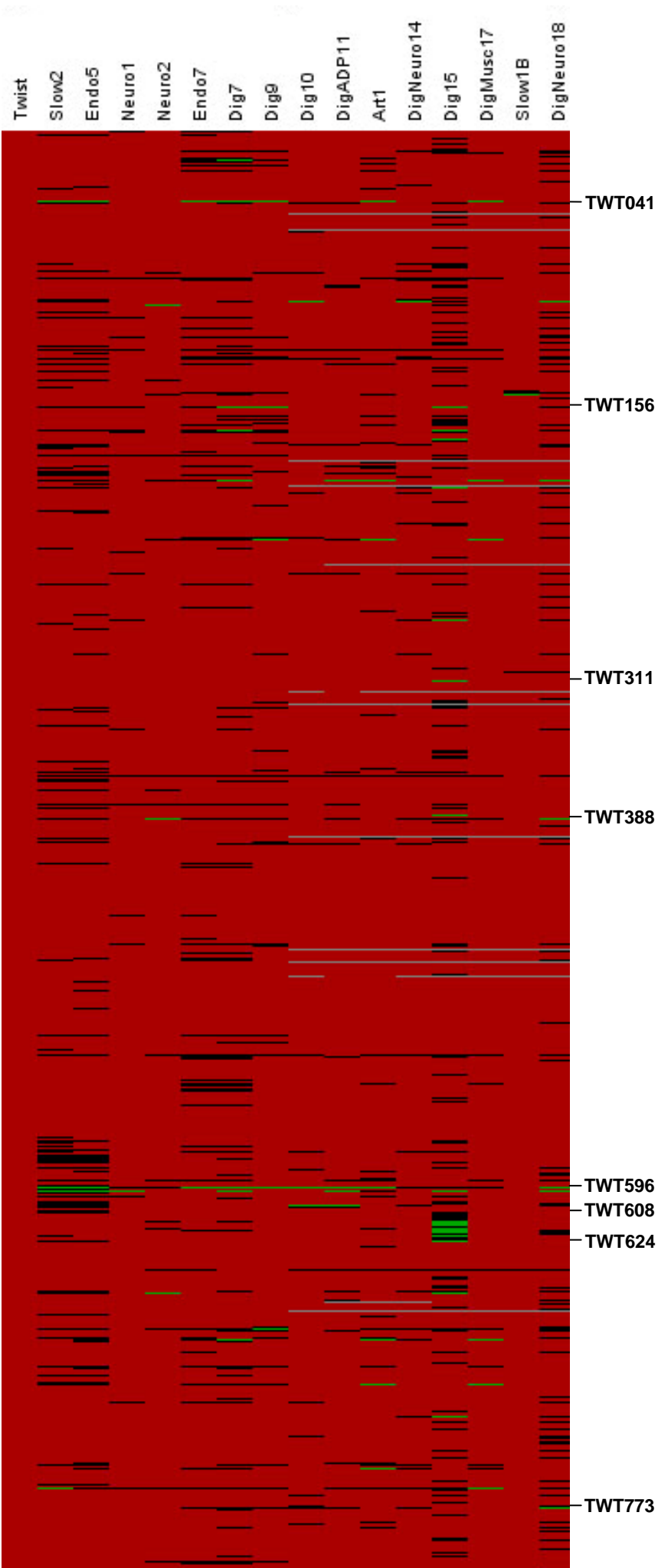

Supplement: Additional file 1 — Genome composition of 16 T. whipplei isolates based on CGH microarray data. This heat map was constructed from the GACK analysis results. Each row corresponds to a specific gene in Twist gene order, whereas columns represent strains analyzed. The CDSs status is color-coded: red, unchanged; green, absent/divergent; black uncertain. The grey lines correspond to missing values. [file 1471-2164-8-349-S1.pdf]
