# Supplementary material for: Comparative genomic analysis of Tropheryma whipplei strains reveals that diversity among clinical isolates is mainly related to the WiSP proteins
Source: BMC Genomics. 2007 Oct 2;8:349. doi: 10.1186/1471-2164-8-349 (PMC2078596; doi:10.1186/1471-2164-8-349)
Supplement: Additional file 2 — Alignment of protein sequences from T. whipplei isolates analyzed in this study. [file 1471-2164-8-349-S2.pdf]

Alignment of protein sequences from several *T. whipplei* isolates

TWT018: deoD
Twist YVLVIGARTHLYQGHGPEAVVHGIKTAHAAGARVAILTNGAGSTVPDWGPGEVVVIX
Dig7 YVLVIGAR**A**HLYQGHGPEAVVHGIKTAHAAGARVAILTNGAGSTVP**E**WGPGEVVVIX

TWT099: unknown
Twist YITYFIVDKLRLYQLPNCLFWLPNCLFWLPNLLLWLV
Dig10 LYHLFH*CRA*VIPVTQPFIVVSPVLGLGLGN*GELFLLHV
DigNeuro14 YITYFIDAGLGYTSYPTIYCG*SCFGLGLGN*GELFL
DigNeuro18 SNRPCIFVPISPISLYHLFH*CRLRLYQLPNHLLWLV

TWT171: unknown
Twist RMHWPVDACWPGIKLFWINWSLLVFSAILLWYAFSACRKVGRVVAYIGSIGGGALLSLRTLLAHPSYTPHL
Dig7 RMHWPVDACWPGIKLF**RT**NWSLLVFSAILLWYAFSACRKV**CH**VVAYIGSIGGGALLSLRTLLAHPSYTPHL
Dig15 RM**Y**GPVDACWPGIKLFW**T**NWSLLVFSAILLWYAFSACRKVG**H**VVAYIGSIGGGALLSLRTLLAHPSYTPHL

TWT176: **unknown**
Twist EVATLFTGAGLGKAAGKAAAKKLFKFLADKAAGKLVKQFLERLVVKAADTAANKVLHFGIDALSLPCKGRKPYYI
Dig15 EVATLFTGAGLGKAAGKAAAKKLFKFLADKAAGKLVKQFLERLVVKAADTAANKVLHFGIDALSLPCKGRKPYYI

TWT199: ftsE
Twist GITTKLLVQDKEVSVFLSTKEGAAGSSDASSCANNITGTGRCDTGLRDT-----HKSDTHNATPRDRALLHDPGLHDTGLQGDVTQGYMT
Dig7 GITTKLLVQDKEVSVFLSTKEGAAG**N**SDASSCANNIT**D**TG**VR**DTG**V**RDT-----HKSDTHNATPRDRALLHDPGLHD**A**GLQGDVTHGYMT
Art1 GTTTKLLVQDKEVSVFLSTKEGAAG**N**SDASSCANNIT**D**TG**VR**DTG**V**RDT**GVRDT**HKSDTHNATPRDRALLHDPGLHD**A**GLQGDVTQGYMT
DigMusc17 GITTKLLVQDKEVSVFLSTKEGAAG**N**SDASSCANNIT**D**TG**VR**DTG**V**RDT-----HKSDTHNATPRDRALLHDPGLHD**A**GLQGDVTQGYMT
DigNeuro18 GITTKLLVQDKEVSVFLSTKEGAAG**N**SDASSCANNIT**D**TG**VR**DTG**V**RDT-----HKSDTHNATPRDRALLHDPGLHD**A**GLQGDV**I**QGYMT
DigADP11 GITTKL**SGSR**KEV**RF**F**CQQ**K**R**--A**QQVTVMQV**A**AQ**IT***PIQGYVIQGYVIPINLIHIMQHPVTAHYYMTLDCMMLDYREM***HKGT***HX**

TWT203: ksgA
Twist PTSQLHGLQVTSGSQHPTNQGVHDTGLRDTHKSDTHNATPRDRALLHDPGLHDTGLQGDVTQGYMT
Dig15 PTSQLHGL------QH**L**TNQG**RC**DTGLRDTHKSDTHNATPRDRALLHDPGLHDTGLQGDVTQG**VRH**

TWT232: WiSP
Twist AVGVFSNGAGPTTPVVVVHPNNRTTVCLTCICNRAVIRLRPKCIGEPVKHILSSX
Dig9 AVGVFSNGA**DS**TTPVVVV**P**PNNRTT**I**CLTCICNR**T**VIRLRPKCIGEPVKHILSSX
Art1 AVGVFSNGA**DS**TTPVVVV**P**PNNRTT**I**CLTCICNR**T**VIRLRPKCIGEPVKHILSSX
DigMusc17 AVGVFSNGA**DS**TTPVVVV**P**PNNRTT**I**CLTCICNR**T**VIRLRPKCIGEPVKHILSSX

TWT277: unknown
Twist IDTNINPYKRRQAPTQHRQAYARAKLTGRRRATNPTARTSQSDRAKLCY
Dig15 IDTN*PA*EATN-PT*HRQAYARAKLTGR**YKPHSSNQPVRSGKTMLS**

TWT311: unknown
Twist LFFVSFGTHLQNLGVKETFMQNKTGLHKFVMLFKQRLWVLGAVLLAIASILQVLSLVFAPLVVVQPLX
Dig15 LFFVSFGTHLQNLGVKETFMQNKTGLHKFVMLFKQRLWVLGAV**PP**AIASILQVLSLVFAPLVVVQPLX

TWT386: trpE
Twist FSKLLDCDPQPWPLGFVGWIDYEVGACLLGVNYPTQQDAPATGQTNLSSWICCNRAICIDYRMGLLKIISFSKNTVDAKEWVX
Dig15 FSKLLD**G**DPQPWPLGFVGW**V**DYEVGACLLGVNYPTQQDAPATGQTNLSSWICCNRAICIDYRMGLLKIISFSKNTVDAKEWVX

TWT388: unknown
Twist STMQGLSEALRVPDKV**T**DTRLFNRNIELMKHGIQSADQAVEKAKESMLLNHKAKKMRAKFDDDVLVLFRLERIAGH
Neuro2 STMQGLSEALRVPDKV**A**DTRLFNRNIELMKHGIQSADQAVEKAKESMLLNHKAKKMRAKFDDDVLVLFRLERIAGH
DigNeuro18 STMQGLSEALRVPDKV**A**DTRLFNRNIELMKHGIQSADQAVEKAKESMLLNHKAKKMRAKFDDDVLVLFRLERIAGH

TWT594: WiSP

```
Twist          VPYL----AVRIVYSVSHTIYSYANPSLKARVLLYTQSQSNVSITPILYGSFSISTYVLVDTTGATSGSTGLPKGLSF-TSGTITGSIDIRX
Slow2          VPYL----AVRIVYSVSHTIYSYANPSLKARVLLYTQSQSNVSITPILYGSFSISTYVLVDTTGATSGXTGLPKGLTLDSSGTITGSIDIRX
Endo5          VRTLLSVLYTQPCYIISRIIYSYANPSLKARVLLYTQSQSNVTITPILYGSFSISTYVLVDTTGATSGSTGLPKGLTLDSSGTITGSIDIRX
Endo7          FRTLLSVLYTQPCYIFSRIIYSYANPSLKARVLLYTQSQSNVSITPILYGSFSISTYVLVDTTGATSGSTGLPKGLSFDSSGTIKGSIDIRX
Dig9           FRTLLSVLYTQPCYIFSRIIYSYANPSLKARVLLYTQNQSNVSITPILYGSFSISTYVLVDTTGATSGSTGLPKGLTLDSSGTITGSIDIRX
Dig10          VPYL----AVRIVYSVSHTIYSYANPSLKARVLLYTQSQSNVSITPILYGSFSISTYVLVDTTGATSGSTGLPKGLTLDSSGTITGSIDIRX
DigADP11       VPYL----AVRIVYSVSHTIYSYANPSLKARVLLYTQSQSNVSITPILYGSFSISTYVLVDTTGATSGSTGLPKGLTLDSSGTITGSIDIRX
Art1           VPYLLSVLYTQPCYIFSRIIYSYANPSLKARVLLYTQSQSNVSITPILYGSFSISTYVLVDTTGATSGSTGLPKGLSFDSSGTIKGSIDIRX
DigNeuro18     FRTLLSVLYTQPCYIFSRTIYSYANPSLKARVLLYTQNQSNVSITPILYGSFSISTYVLVDTTGATSGSTGLPKGLSF-TSGTITGSIDIRX
Dig7           SVPCYPCYILSHAIYSAVSYTHTLIQA*KHVYYCTHRVRVM*VLHLYSTEAFLYPHMY**IQQVLHKGSTGLPKGLSFDSSGTIKGSIDIRX
```

TWT596: WiSP

```
Twist          ANGSDTFLYPTDQLTEGGEGYTPIPVSTQSLSNFTIDTVSNTWTYTGSGKTNQTQGTVTLIITAYKNSTSSTQQTW
Dig7           QMAKRHTYTPQIN*PRVEKSYTPITVSTQSLH-FTIDLTSNTWTYTGGGNTNQTQGTVTLTITAYKNSSTSTQRTW
```

TWT604: unknown

```
Twist          LLLRNLRYRLGHRLCHLVGRPRCLWVIVGGSLFLLIVFSKIRRENIQQNKIX
Dig10          LLLRNLRYRLGHRLGHRVGRPRCLWVIVGGSLFLLIVFSKIRRENIQQNKIX
```

TWT653: membrane protein

```
Twist          TVTLTHVTQQTRNSTDRWVIGVTDLYYGTDAWGFYLKAGWSLQAALVAVTSVVPSGVPLKAALRVILPALLKGAVKFAGS
Neuro2         L*PLHMQHNRHRQDFPGVMTHGVST*NWNGMNKLPLLQALLPLVAVPAWPLRPL*K*P*RQAPRFWLAWRVVDKLSDIIGA
```

TWT673: unknown

```
Twist          RPAAVSVAGGLTGFVGGAVSGCVLGGRLVDDLVNGFNAGFNAVGDFVGKLFG
Dig9           RPAAVSVAGGLTGFVGGTVSGCVLGGS**SCQWV*CWI*RRW*LCRKAIRI
```

TWT679: unknown

```
Twist          KPLSRGIKIGASVVALCVSVLLIAIFSLMTRPIGNDYHTTSFKVKSDNEVIVEFSFTGHIPTVCAVRVLGRDMSTVGWKVVPVLSPHMEVGLRTTKVAX
Dig7           KPLSRGIKIGASVVAICVSVLLIGIFSLMTRPIGNDYHTTSFKVKSDNEVIVGFSFTGHIPTVCAVRVLGHDMSTVGWKVVPVLSPHMEVGLRTTKVAX
Art1           KPLSRGIKIGASVVAICVSVLLIGIFSLMTRPIGNDYHTTSFKVKSDNEVIVGFSFTGHIPTVCAVRVLGHDMSTVGWKVVPVLSPHMEVGLRTTKVAX
DigMusc17      KPLSRGIKIGASVVAICVSVLLIGIFSLMTRPIGNDYHTTSFKVKSDNEVIVGFSFTGHIPTVCAVRVLGHDMSTVGWKVVPVLSPHMEVGLRTTKVAX
```

TWT704: unknown

```
Twist          YKTYFSRTRWERELSEAVCFNGKQLNSAIHYGIYRTGVIDPVWTAFIFKWVYAFR
Art1           YKTYFSRTRWERELSEAVCFGGKQLNSAINYGVYRTGVIDPVWTVFIFKWVYAFR
DigMusc17      YKTYFSRTRWERELSEAVCFGGKQLNSAINYGVYRTGVIDPVWTVFIFKWVYAFR
```

TWT751: unknown

```
Twist          IRKASQMLKDWRLSGLTLFVTMEPCTMCAGAIVTSRISRVVFGAFNNKTGSVGSRIDILR
Art1           IRKASQMLKDWRLSGLTLFVTMEPCTMCAGAIVTSRISRVVFGVFNNKTGSVGSRIDILR
```

TWT762: unknown

```
Twist          LRGWSGSGDFGVSRLTRVLPLVLPVALVVEEGASERLPNNFLNGHVMPCHIHLNGAAIPLPTFDQNPPPLLSVGVERHLSNIDCX
DigMusc17      LRGCSGSCDFCAPRLTRFLPLVLPVALVVEEGASERLPNNFLNGHVMPCHIHLNGAAIPLPTFDQNPPPLLSVGVERHLSNIDCX
```

TWT773: unknown

```
Twist          IAHLTLLTATLLTATLSALGIEKQHGLSLERASTWRSALRNLGIKQQINMGFVPGHMLWHAGHLV
DigNeuro18     IAHLTLLSAN-----LSALGIEKRHGLSLERASTWRSALRNLGIKHTNKHGVRARSHAMACWASGL
```
