# Supplementary material for: Comparative genomic analysis of Tropheryma whipplei strains reveals that diversity among clinical isolates is mainly related to the WiSP proteins
Source: BMC Genomics. 2007 Oct 2;8:349. doi: 10.1186/1471-2164-8-349 (PMC2078596; doi:10.1186/1471-2164-8-349)
Supplement: Additional file 3 — Nucleotide sequences of the genomic region amplified in T. whipplei DIG15 with primers TWT607F1 and TWT625R1. [file 1471-2164-8-349-S3.pdf]

Nucleotidic sequences of the genomic region amplified in *T. whipplei* DIG15 with primers TWT607F1 and TWT625R1

```
CGCCAATTACCAGTGAAATAATTACGAATAATACCTGACAAACTTGAATTATTTGAATTA
GATCCCGAACCGGAGGATGAAGCTGGCTTTTTGACTAATTTACCATAAAGATAAAACGGC
AAGAAGTAAACACCTCCAACAGAAACAGCAGACATAAAAGCTCGGAAAAACTTACCACCC
CAGCCAGAAAAACTAGGTAAATGGGCAGATATCTTTCTATACAAGGCCTTAATGCCAACT
TGCCCATATAGAGATGCTACTGGATAGGCATAGCTTTTCCAGTCATAACATGCATCTGAA
TTATGTGCTGCAGAGTAAAAAGCATTGTAGGCATTAACCAGTGTTGTTACTGCGGCAGGT
AGGAGGGCGCCGGCAACAGCACCAAGGAAACCTTGGCTTGTTGTGTGGTGGACTGGGGTG
CAAGTGTGAGAGAACCAACTGTATATAGCTTTGAATGGCTTGGTTAGATAGTATTTGACC
CTTTGGAAGAATTTCATACTACTTAGCACAAGGGTATATACAGCAGTAACAGTTGTGCTA
CCAAGGGTTGTAGAGGAAGTATAGGAAGGTGCAGTTGCAGTAATAGTTACTGTATATGTT
CCAGGCACAATACTTGGGGTGATTGTTATATTGCCATTGGTTTTATCAATATTGATGGTT
ACGGTGTTACTGTCTGTGGTTAGGGTGGTTGGGCTTATTGTGTAACCGGTTATAAAACCT
GTTACAGATAAGCTGACAGGTGTTGTTGTGTATATGGTTATGGTTGTACCAGATAGATAT
GGTGCTGTACTGGTAACAAGAAATGTATATGTTGCACTAACAATTGATGTTTTTGATGCA
GTGGCAGTTACTACTACTGTATATATACCTTGGCTAACACCTGTATCTATACTTCCTGTT
ATCTCTCCTTTGGTTTTATCAAGGGTTAGTCCTTTAGGTAGACCTGTAGCACCAGTTCCA
CCAGATGTAGCATCTGTAGGTGTAGCTATATAGCCAGATAGAGAACCTCTTGCGTTGGGT
GTAATAGTTATATTAGTCTGACCTATAGATACACCTGTGGCAGATAGAGATGTACCAAAG
AGGGAGCCTATGCCACTGAATAGGTTGCCCAAGATGCTGCCGGGTGATCGTGTGGGGGCA
GCTCTCTTAGATCTGTTATGGGTTGTGGGGTTGTGTGTATCTATTGCATAGGTGATATGG
GTGGTTGTGTCTTTGTTTATGGCTATGGCTGTTATGGGAGATGTTGGTAGGGATGAGAGA
TGTATCTGTCCGGTGTATCTATCTATGGTTATGTCTTTGTTTGGGTGTGTTAGGGAGAAG
GTGTATCTGGAGTTTCTGAGTGATGGTATGTCTGAGAGATATGTGTTATCTATCTGTTTG
TGTATGGGAACAGTTATCTTTGGTTTCTCTGTTACATGTATGTCAAAGAAGAAGATGTTA
CGGGTTGTTAGGAGAGCTATGAGATTGGTTGTTGTCTTTGTTATGGAGACTACTTTCTCA
TCTCCGTATGGGGTTGCTATGTCTGTATGAGGGAAGGTTAGGAAAGATGATGGATCTGCT
TTCTTATCTGTGTAATAGAGGGATATATGTGTGTTGGTTATATATGGGACATATAGGTAT
GAGGATGTATCTGTGTATGGGACATATATATGTGTCTGTGGTTGGGGTGAGAGAGATGGG
GTGCAGGACAGGGTTTGTTTTGTACTGCTTGTAACAGGATCTATTAGGAGAGATACATTT
GTGTTAGTTGTTACACATACTTCTTGTGTATTTGTATTACTTACTCTTGTGTATTTGGTA
CTAGATGAAACAGATGTAGATGAAACAGATGATAGGAGGGATAGGGCAGGAGGTGTCTGT
CTGGCAAAGGCTGTTTCATATTGTAGGGATGATAGGAGGAGAAGGGTTAATAGTAAGGTA
AAGGCTTTGGTGAGGTAAGAGAGAGGGTTAGGCGATGTTGGCAATCCCCCCCCCCTTCTG
TTTTGTTTTCTGTTTTGGGGGGTTACCCTATTAGGGACCAGTTTGCACTTCATAATACTC
ATAGTACCTCACTATACATGCGTCTGCATACACGCACCTGTGCCGATTGTAACACATTTG
```
