# Supplementary material for: Comparative genomic analysis of Tropheryma whipplei strains reveals that diversity among clinical isolates is mainly related to the WiSP proteins
Source: BMC Genomics. 2007 Oct 2;8:349. doi: 10.1186/1471-2164-8-349 (PMC2078596; doi:10.1186/1471-2164-8-349)
Supplement: Additional file 4 — Oligonucleotide primers used for PCR and sequencing. [file 1471-2164-8-349-S4.pdf]

Oligonucleotide primers used for PCR and sequencing

Gene ID	Primer sequence (5' --> 3') for PCR		Product size (bp) in Twist	Primer sequence (5' --> 3') for sequencing	
TWT018	TWT017F1	ccggatcattctcaatccacaagc	990	TWT018sF1	tcctgcccgatagatcttgcccgat
	TWT019R1	tcttgacagacactctcagccttct		TWT019sR1	tcttgacagacactctcagccttct
TWT041	TWT040F1	aggtaaagcgatccattgggcag	1380	TWT041sF1	gcgctgttagcggtgcagtt
	TWT042R1	agggttacccagatcaagcgagaac		TWT041sR1	caggacacgtaaacgcaaga
TWT099	TWT098F1	tctttcatagaccggccagggctc	2460	TWT099sF1	cgtgagttctatataacgtg
	TWT100R1	aaagctggatccgtatctgaatgtt		TWT099sR1	gtggcagcggctttagctgg
TWT101	TWT100F1	ccgctgccacaaggcccttgaac	4498	TWT101sF1	cggcaaaaagcgcgtcaaacg
	TWT102R1	gcaaaaaacgcgttagcgcggagc		TWT101sR1	gcatgaatttctatgcatacct
TWT151	TWT150F1	ctgcccaatagagttttgcccaatg	1920	TWT151sF1	gccacacattcatcctctac
	TWT152R1	gatgtgcgtgttatgcgtgttcgaa		TWT151sR1	ggtaaaatcctgcgcgaata
TWT158	TWT157F1	cggctaaaatccgacagcaaatgac	1740	TWT158sF1	gggtcatgtgatgacattcg
	TWT159R1	cccaagtgacctgcgaaagttagc		TWT158sR1	cacggtccacatttcacaag
TWT171	TWT170F1	gagtggaacattgaatctgttcaccg	4800	TWT171sF1	gccacgttaattgcttcata
	TWT172R1	cagcaatagacaggatggttcgtatc		TWT171sR1	aggcgaataagcttgggttt
TWT176	TWT175F1	aaggcaacctcctgggacatctgac	1560	TWT175sF2	gtacactttaatcccgtggggtact
	TWT177R1	gcactcgggaagacgctcgtctgtg		TWT177sR2	acggccaattaagactaacgaagcg
TWT199	TWT198F1	gccggaataaaatggcacgcgc	3060	TWT199sF1	cgagttgtcgaggtcagtca
	TWT200R1	aaatgtgacgataatcacagaggag		TWT199sR1	ctgtatctgccgactggaca
TWT203	TWT202F1	atacaggaacatataccttccctg	1620	TWT203sF1	cgtgcacaggtcaatctcat
	TWT204R1	gagacaacctcgcatcctcgacc		TWT203sR1	ccgctctgatcgtcactgta
TWT232	TWT231F1	catcagcccgcattcttcatcttc	1020	TWT232sF1	ggtctacatattacgtagact
	TWT233R1	gcaattaagggtgctatttttgc		TWT232sR1	cgcagaagaatcgagggcaa
TWT277	TWT276F1	caagcttcaaagaagcacgtctgag	1140	TWT277sF1	gcggttattctcgtttgtcc
	TWT278R1	gtatgctcagcgcctgtcgacatca		TWT277sR1	cagcctggctttttatggtt
TWT311	TWT310F1	aaacatatctcgtacaatttcctg	1380	TWT311sF1	gtattttgccaccgctgatt
	TWT312R1	gaatttggccgcaccattaacttc		TWT311sR1	cgctgattttaggccatcag
TWT386	TWT385F1	ggggcccatttttcttctaaggc	1620	TWT386sF1	ggcatgagttttgagtttgct
	TWT387R1	caattaggagcagtgggttaatgc		TWT386sR1	cgattcggagaattgctttt
TWT388	TWT387F1	ttgccgcatggaacacgtttgttg	1740	TWT388sF1	gcggttggatttgtggtaaa
	TWT389R1	gtggaaaaatgcaagacttgtaac		TWT388sR1	ggcggccttattcaactgt
TWT594	TWT593F1	tccatggaggatgcgaaatagttg	720	TWT594sF1	ggcgattggatctgtctatctt
	TWT595R1	gaggctattaggattcgtaagattg		TWT594sR1	gaccggatgagtgtgttgag
TWT596	TWT595F1	tgtatagggccggatcgtttatc	2040	TWT596sF1	caggtcggtgcaggtatagg
	TWT597R1	cagagataaacgcagatcttggag		TWT596sR1	cacccaagacaacgggtta
TWT604	TWT603F1	cgagacgaggccatcatttataccaa	2700	TWT604sF1	gcacctcttgtcttgggtgt
	TWT605R1	tgtgtgcaccccagtccacctcgc		TWT604sR1	cccatgatacccatctggtc
TWT653	TWT652F1	gccctgccaagcacagttaacgg	1975	TWT653sF1	gccacacttggttctgaagg
	TWT654R1	cattatgcgtagagccaatgcccttc		TWT653sR1	cccacatacgcaacaacatc
TWT673	TWT672F1	gcgcctgagcatttaactgtgatac	600	TWT672sF1	gcgcctgagcatttaactgtgatac
	TWT674R1	cgacactgccggttaagggatggcg		TWT674sR1	cgacactgccggttaagggatggcg
TWT679	TWT678F1	gcctccgacctttccggaccaac	1380	TWT679sF1	cctggatgaatcgtgaaaca
	TWT680R1	gctatttctctgttccccagcagg		TWT679sR1	ccttcagaccttgcttgctc

TWT704	TWT703F1	ttcgctatttgttacaacggtg	420	TWT704sF1	ggtgacaagctcccatcct
	TWT705R1	gaaagctagtgccggtaccattcctg		TWT704sR1	ccattcctgacgcaagtaca
TWT722	TWT721F1	cgatgatgtggccgctgctgtggag	1140	TWT721sF1	cgatgatgtggccgctgctgtggag
	TWT723R1	gacccgattccgttatgaatggcgt		TWT723sR1	gacccgattccgttatgaatggcgt
TWT751	TWT750F1	ctccgatttgttttgtccagtctgtg	1320	TWT751sF1	ggtgattgccgatgtgaac
	TWT752R1	gtgtgaccgttatgtttgcgtcggg		TWT751sR1	tgcctaagttcggttttcca
TWT762	TWT761F1	ctcgttacagtgtatgtgagaac	2740	TWT762sF1	gcaagtgtgagagaaccaagtg
	TWT763R1	ctccaaatgcactccatagcttg		TWT762sR1	caggcattacccctgcttt
TWT773	TWT772F1	caatccgtcatcataggccgggga	660	TWT772sF1	caatccgtcatcataggccgggga
	TWT774R1	agcatcctcggttgcttgattggta		TWT774sR1	agcatcctcggttgcttgattggta
TWT607/625	TWT607F1	cgccaattaccagtgaaataattacg	21 088	TWT607sF1	cgccaattaccagtgaaataattacg
	TWT625R1	tactcatagtacctcactatacatg		TWT607sF2	ctttggaagaatttcatactacttag
				TWT607sR1	ctacaatgctttttactctgcagcac
				TWT607sR2	tataaccagttacgcaataaaccca
				TWT608sF1	tttaggtagacctgtagcattagt
				TWT608sF2	tatctatctgtttgtgtatgggaac
				TWT608sF3	caggaggtgtctgtctggcaaagg
				TWT608sR1	tctaagagagctgccaccatacgat
				TWT608sR2	gatacatcctcatacctatatgtcc
				TWT608sR3	gctagaattgccacacactgataga
				TWT624sF1	ctctgttacatgtatgtcaaagaag
				TWT624sR1	tctcctaatagatcctgttacaagc
				TWT625sF1	tactcatagtacctcactatacatg
				TWT625sF2	gaaaataaccccgcagaggcaattg
				TWT625sR1	gtatctgcgggtaatggcatgtccg
				TWT625sR2	gggaattcgggtggtcctcttgttg
				TWT625sR3	cgcccaggtggtaccccggtgccta
